# Supplementary material for: Fatty Acid Sensing in the Gastrointestinal Tract of Rainbow Trout: Different to Mammalian Model?
Source: Int J Mol Sci. 2023 Feb 21;24(5):4275. doi: 10.3390/ijms24054275 (PMC10002231; doi:10.3390/ijms24054275)
Supplement: Supplementary file 1 [file ijms-24-04275-s001.zip › ijms-2162866-supplementary.pdf]

Supplementary Figure S1. Full-length images of Western blots included in Figure 4H. Bands shown in Figure 3 are boxed. For each protein, two separate blots containing in total of six samples per treatment were performed. Molecular markers are included. See corresponding figure legend for details.

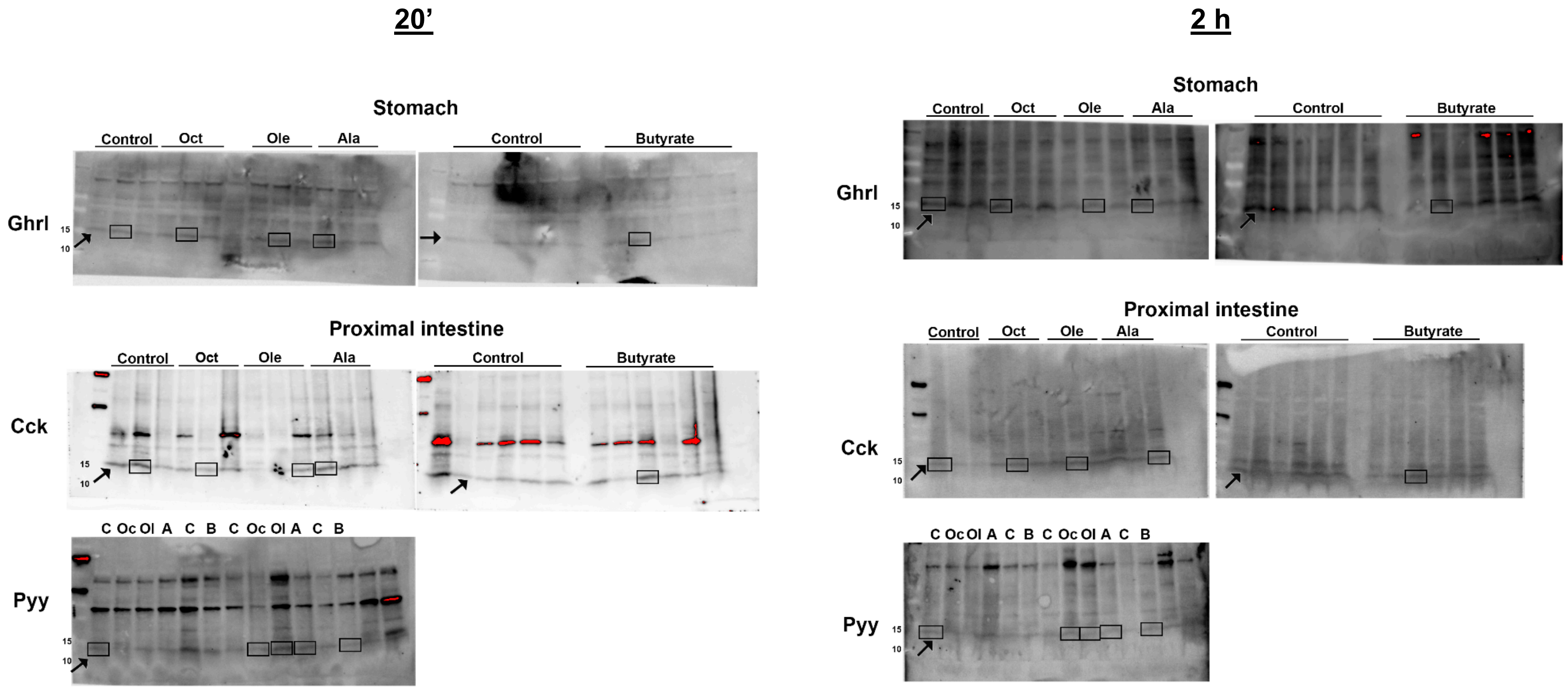

Supplementary Figure S2. Schematic representation of the rainbow trout gastrointestinal tract, showing the regions in which it was divided for the experiments carried out in this study. The areas sampled within each region are squared. For the tissue distribution study, all squared regions were sampled. For the study of the effects of luminal fatty acids on sensing mechanisms, sampling from middle intestine was restricted to the intermediate portion.

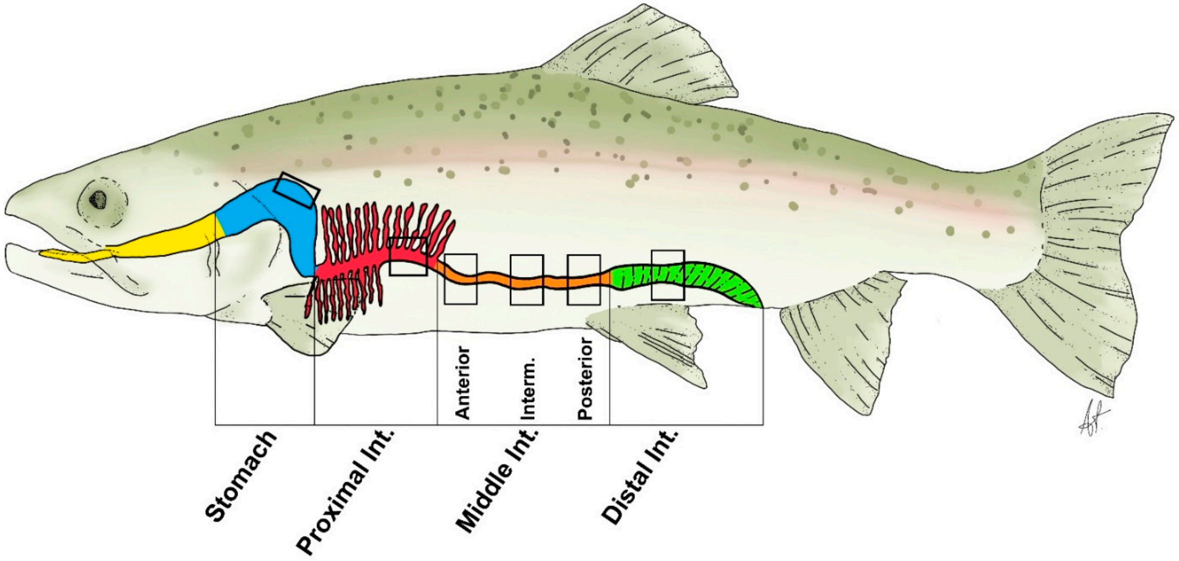

Supplementary Table S1. Abundance of mRNAs encoding intracellular signaling elements in rainbow trout stomach and intestine (proximal, middle and distal) 20 min and 2 h after intragastric administration of vehicle alone or containing octanoate, oleate, ALA or butyrate. Data obtained by RT-qPCR were normalized to the expression of *actb* and *ee1af1α* and is expressed as mean + SEM (n = 6), relative to the control group. Asterisks indicate significant ~~Statistical~~ differences (p<0.05) between treatment and control groups as assessed by one-way ANOVA followed by Dunnett's test ~~were assessed by *t*-test. * p<0.05, ** p<0.01, *** p<0.001~~.

gnai1:

	20 min					2 h				
	CONTROL	OCT	OL	ALA	BUT	CONTROL	OCT	OL	ALA	BUT
Stomach	1.00 ± 0.05	1.12 ± 0.02	0.96 ± 0.03	0.99 ± 0.18	1.40 ± 0.02*	1.00 ± 0.07	0.95 ± 0.15	0.94 ± 0.09	1.34 ± 0.07*	0.88 ± 0.09
Prox. Int.	1.00 ± 0.05	1.07 ± 0.13	1.05 ± 0.12	1.42 ± 0.13*	1.01 ± 0.07	1.00 ± 0.05	0.97 ± 0.10	1.27 ± 0.09*	1.35 ± 0.11*	1.07 ± 0.18
Middle Int.	1.00 ± 0.08	1.22 ± 0.05*	1.02 ± 0.08	1.05 ± 0.09	0.74 ± 0.08*	1.00 ± 0.11	1.24 ± 0.11	1.76 ± 0.16*	2.03 ± 0.23*	1.01 ± 0.07
Distal Int.	1.00 ± 0.19	1.77 ± 0.08*	1.24 ± 0.15	1.70 ± 0.20*	1.11 ± 0.13	1.00 ± 0.08	0.97 ± 0.07	1.15 ± 0.08	1.31 ± 0.11*	0.72 ± 0.07*

plcb1:

	20 min					2 h				
	CONTROL	OCT	OL	ALA	BUT	CONTROL	OCT	OL	ALA	BUT
Stomach	1.00 ± 0.06	0.88 ± 0.06	1.03 ± 0.08	1.20 ± 0.20	1.37 ± 0.13*	1.00 ± 0.07	0.76 ± 0.06*	0.90 ± 0.07	1.37 ± 0.15*	1.77 ± 0.26*
Prox. Int.	1.00 ± 0.12	0.88 ± 0.10	0.96 ± 0.12	0.95 ± 0.07	2.45 ± 0.13*	1.00 ± 0.09	0.73 ± 0.06*	1.79 ± 0.29*	1.51 ± 0.14*	0.90 ± 0.04
Middle Int.	1.00 ± 0.10	1.12 ± 0.08	1.35 ± 0.11*	1.69 ± 0.14*	1.79 ± 0.19*	1.00 ± 0.07	1.10 ± 0.12	1.22 ± 0.05*	1.30 ± 0.12*	0.89 ± 0.07
Distal Int.	1.00 ± 0.11	0.98 ± 0.05	0.55 ± 0.05*	0.80 ± 0.09	1.42 ± 0.19	1.00 ± 0.08	0.73 ± 0.05*	0.99 ± 0.04	1.08 ± 0.08	1.03 ± 0.12

plcb3:

	20 min					2 h				
	CONTROL	OCT	OL	ALA	BUT	CONTROL	OCT	OL	ALA	BUT
Stomach	1.00 ± 0.11	0.91 ± 0.13	0.92 ± 0.05	1.01 ± 0.06	0.53 ± 0.04*	1.00 ± 0.08	0.88 ± 0.08	1.05 ± 0.08	1.03 ± 0.06	1.11 ± 0.07
Prox. Int.	1.00 ± 0.08	0.93 ± 0.09	0.93 ± 0.11	0.84 ± 0.05	0.88 ± 0.08	1.00 ± 0.06	0.85 ± 0.08	1.50 ± 0.08*	1.30 ± 0.08*	1.05 ± 0.07
Middle Int.	1.00 ± 0.09	1.06 ± 0.05	1.17 ± 0.06*	1.26 ± 0.08*	0.58 ± 0.06*	1.00 ± 0.06	0.96 ± 0.06	1.20 ± 0.06*	1.11 ± 0.06	1.00 ± 0.10
Distal Int.	1.00 ± 0.14	1.09 ± 0.06	0.97 ± 0.10	0.90 ± 0.05	0.53 ± 0.06*	1.00 ± 0.08	1.02 ± 0.11	1.16 ± 0.06	1.08 ± 0.04	1.17 ± 0.06

plcb4:

	20 min					2 h				
	**CONTROL**	**OCT**	**OL**	**ALA**	**BUT**	**CONTROL**	**OCT**	**OL**	**ALA**	**BUT**
Stomach	1.00 ± 0.08	0.68 ± 0.05*	0.62 ± 0.05*	0.69 ± 0.08*	0.52 ± 0.05*	1.00 ± 0.04	0.72 ± 0.08*	0.71 ± 0.06*	0.58 ± 0.06*	1.00 ± 0.10
Prox. Int.	1.00 ± 0.09	0.89 ± 0.08	0.76 ± 0.07*	0.63 ± 0.06*	0.92 ± 0.06	1.00 ± 0.10	1.10 ± 0.12	1.41 ± 0.21	1.60 ± 0.09*	0.98 ± 0.11
Middle Int.	1.00 ± 0.08	1.23 ± 0.13	1.25 ± 0.08*	1.61 ± 0.17*	0.78 ± 0.09	1.00 ± 0.07	1.09 ± 0.08	1.30 ± 0.07*	1.38 ± 0.17*	0.71 ± 0.06*
Distal Int.	1.00 ± 0.13	1.08 ± 0.15	0.43 ± 0.02*	0.79 ± 0.02*	1.68 ± 0.20*	1.00 ± 0.08	0.80 ± 0.04*	0.78 ± 0.09*	0.73 ± 0.04*	1.11 ± 0.10

itpr1:

	20 min					2 h				
	**CONTROL**	**OCT**	**OL**	**ALA**	**BUT**	**CONTROL**	**OCT**	**OL**	**ALA**	**BUT**
Stomach	1.00 ± 0.15	1.03 ± 0.16	0.94 ± 0.08	0.97 ± 0.08	0.97 ± 0.10	1.00 ± 0.07	0.97 ± 0.10	0.85 ± 0.08	0.91 ± 0.08	1.08 ± 0.13
Prox. Int.	1.00 ± 0.14	1.23 ± 0.13	1.25 ± 0.19	0.87 ± 0.07	1.72 ± 0.23*	1.00 ± 0.06	1.15 ± 0.07	1.08 ± 0.10	2.69 ± 0.25*	1.37 ± 0.13*
Middle Int.	1.00 ± 0.15	1.78 ± 0.21*	1.82 ± 0.21*	2.31 ± 0.13*	2.19 ± 0.05*	1.00 ± 0.08	1.14 ± 0.11	1.23 ± 0.12	1.41 ± 0.07*	1.03 ± 0.14
Distal Int.	1.00 ± 0.13	1.47 ± 0.14*	1.14 ± 0.12	1.46 ± 0.10*	1.10 ± 0.21	1.00 ± 0.09	0.96 ± 0.12	0.98 ± 0.10	0.95 ± 0.04	1.46 ± 0.16*

itpr3:

	20 min					2 h				
	**CONTROL**	**OCT**	**OL**	**ALA**	**BUT**	**CONTROL**	**OCT**	**OL**	**ALA**	**BUT**
Stomach	1.00 ± 0.12	0.60 ± 0.06*	0.63 ± 0.07*	0.56 ± 0.07*	0.41 ± 0.04*	1.00 ± 0.11	0.62 ± 0.06*	0.60 ± 0.05*	0.57 ± 0.06*	1.25 ± 0.14
Prox. Int.	1.00 ± 0.12	0.63 ± 0.05*	0.70 ± 0.11	0.59 ± 0.08*	0.84 ± 0.03	1.00 ± 0.04	1.17 ± 0.07	0.99 ± 0.02	1.10 ± 0.05	1.00 ± 0.13
Middle Int.	1.00 ± 0.10	1.05 ± 0.11	1.06 ± 0.08	1.06 ± 0.12	0.74 ± 0.05	1.00 ± 0.08	1.06 ± 0.07	1.30 ± 0.07*	1.42 ± 0.04*	0.92 ± 0.08
Distal Int.	1.00 ± 0.12	1.19 ± 0.11	0.93 ± 0.16	0.75 ± 0.08	0.57 ± 0.04*	1.00 ± 0.08	0.89 ± 0.09	0.96 ± 0.04	0.82 ± 0.05	1.04 ± 0.07

ac:

	20 min					2 h				
	**CONTROL**	**OCT**	**OL**	**ALA**	**BUT**	**CONTROL**	**OCT**	**OL**	**ALA**	**BUT**
Stomach	Not detectable					Not detectable				
Prox. Int.	1.00 ± 0.15	1.32 ± 0.31	0.99 ± 0.20	1.00 ± 0.09	0.71 ± 0.10*	1.00 ± 0.12	0.88 ± 0.08	1.02 ± 0.08	0.84 ± 0.11	1.07 ± 0.06
Middle Int.	1.00 ± 0.21	1.29 ± 0.25	1.33 ± 0.13	1.30 ± 0.22	0.51 ± 0.04*	1.00 ± 0.16	1.07 ± 0.06	1.44 ± 0.16*	1.17 ± 0.13	0.51 ± 0.04*
Distal Int.	1.00 ± 0.13	1.07 ± 0.11	1.04 ± 0.17	1.30 ± 0.04*	0.98 ± 0.05	1.00 ± 0.15	1.18 ± 0.25	0.88 ± 0.17	0.85 ± 0.13	0.81 ± 0.11

Supplementary Table S2. Effects of intragastric administration of 50 µmol/mL of octanoate, oleate, ALA or butyrate on the plasma levels of glucose, lactate, triglycerides and fatty acids in rainbow trout at 20 min and 2 h post-administration. Data are expressed as mean + SEM (n = 12). ~~Different letters indicate significant~~Statistical differences were assessed by ANOVA and Student-Newman-Keuls test (p<0.05) and none were found.

Glucose (mmol/L):

20 min					2 h				
**CONTROL**	**OCT**	**OL**	**ALA**	**BUT**	**CONTROL**	**OCT**	**OL**	**ALA**	**BUT**
61.84 ± 4.90	53.04 ± 6.20	52.35 ± 8.14	59.18 ± 3.96	69.30 ± 13.52	58.74 ± 2.93	66.47 ± 4.70	64.77 ± 6.54	64.40 ± 6.96	62.20 ± 4.35

Lactate (mmol/L):

20 min					2 h				
**CONTROL**	**OCT**	**OL**	**ALA**	**BUT**	**CONTROL**	**OCT**	**OL**	**ALA**	**BUT**
34.93 ± 1.11	28.74 ± 4.39	29.26 ± 6.27	39.39 ± 5.64	36.68 ± 1.09	29.63 ± 4.46	27.04 ± 3.90	31.72 ± 3.95	29.71 ± 4.90	28.74 ± 2.38

Triglycerides (mmol/L):

20 min					2 h				
**CONTROL**	**OCT**	**OL**	**ALA**	**BUT**	**CONTROL**	**OCT**	**OL**	**ALA**	**BUT**
71.04 ± 5.10	77.08 ± 8.34	78.69 ± 9.20	72.58 ± 6.19	59.96 ± 16.32	62.97 ± 4.40	47.053 ± 3.80	62.90 ± 5.044	73.1 ± 4.92	66.53 ± 4.63

Fatty acids (mmol/L):

20 min					2 h				
**CONTROL**	**OCT**	**OL**	**ALA**	**BUT**	**CONTROL**	**OCT**	**OL**	**ALA**	**BUT**
0.14 ± 0.02	0.34 ± 0.05	0.19 ± 0.02	0.21 ± 0.02	0.15 ± 0.01	0.14 ± 0.02	0.20 ± 0.02	0.18 ± 0.03	0.18 ± 0.01	0.14 ± 0.01
